# SARS-CoV-2 Monitoring in Wastewater Reveals Novel Variants and Biomarkers of Infection

**DOI:** 10.3390/v14092032

**Published:** 2022-09-13

**Authors:** Jenna McGowan, Monica Borucki, Hicham Omairi, Merina Varghese, Shahnaz Vellani, Sukanya Chakravarty, Shumin Fan, Srestha Chattopadhyay, Mashuk Siddiquee, James B. Thissen, Nisha Mulakken, Joseph Moon, Jeffrey Kimbrel, Amit K. Tiwari, Roger Travis Taylor, Dae-Wook Kang, Crystal Jaing, Ritu Chakravarti, Saurabh Chattopadhyay

**Affiliations:** 1 Physiology and Pharmacology, University of Toledo College of Medicine and Life Sciences, Toledo, OH 43614, USA; 2 Physical and Life Sciences Directorate, Lawrence Livermore National Laboratory, Livermore, CA 94550, USA; 3 Department of Civil and Environmental Engineering, University of Toledo College of Engineering, Toledo, OH 43607, USA; 4 Medical Microbiology and Immunology, University of Toledo College of Medicine and Life Sciences, Toledo, OH 43614, USA; 5 College of Pharmacy and Pharmaceutical Sciences, University of Toledo, Toledo, OH 43606, USA; 6 Computing Directorate, Lawrence Livermore National Laboratory, Livermore, CA 94550, USA; 7 Center for Medical Bio-Allied Health Sciences Research, Ajman University, Ajman P.O. Box 346, United Arab Emirates

**Keywords:** wastewater-based epidemiology, SARS-CoV-2, surveillance, COVID-19, biomarkers

## Abstract

Wastewater-based epidemiology (WBE) is a popular tool for the early indication of community spread of infectious diseases. WBE emerged as an effective tool during the COVID-19 pandemic and has provided meaningful information to minimize the spread of infection. Here, we present a combination of analyses using the correlation of viral gene copies with clinical cases, sequencing of wastewater-derived RNA for the viral mutants, and correlative analyses of the viral gene copies with the bacterial biomarkers. Our study provides a unique platform for potentially using the WBE-derived results to predict the spread of COVID-19 and the emergence of new variants of concern. Further, we observed a strong correlation between the presence of SARS-CoV-2 and changes in the microbial community of wastewater, particularly the significant changes in bacterial genera belonging to the families of *Lachnospiraceae* and *Actinomycetaceae*. Our study shows that microbial biomarkers could be utilized as prediction tools for future infectious disease surveillance and outbreak responses. Overall, our comprehensive analyses of viral spread, variants, and novel bacterial biomarkers will add significantly to the growing body of literature on WBE and COVID-19.

## 1. Introduction

Severe acute respiratory syndrome (SARS)-coronavirus (CoV)-2 infection is the causative agent for the ongoing COVID-19 pandemic, which began in 2019 and resulted in extensive damage around the world [1,2,3,4,5,6]. SARS-CoV-2 is a member of the *Coronaviridae* family, containing a positive-sense, single-stranded RNA genome [7,8,9,10,11]. Other members of the family, such as SARS-CoV, Middle East respiratory syndrome (MERS)-CoV, and human CoVs, cause respiratory infection in humans. Human CoV (e.g., NL63) infection causes the common cold, and SARS-CoV infection caused major respiratory infections in 2003. Although not a pandemic, the emergence of MERS-CoV resulted in hundreds of fatal infections [7,9,10,11]. SARS-CoV-2 infects and replicates in epithelial cells in the upper and lower respiratory system, resulting in extensive lung damage [1,2,3,4,6,12,13,14,15]. Although the initial site of infection is the respiratory system, SARS-CoV-2 infection also causes damage to secondary organs, e.g., heart, liver, brain, and others [1,2,3,5,6,12,13,15,16,17,18,19].

SARS-CoV-2 primarily replicates in the lungs and their associated cells, e.g., epithelial cells and macrophages; however, viral particles can also be shed through the feces and urine of infected individuals [20,21]. The analyses of the feces and urine of infected individuals revealed the presence of viral gene fragments, though it is not clear whether infectious virus particles were shed [22,23,24,25,26,27]. Household and community sewer system pipes deliver the viral gene fragments to wastewater treatment plants (WWTPs), where they accumulate in the raw sewage. Raw sewage, collected from WWTPs, therefore, is a great source of viral gene fragments [23,24,25,28]. The analysis of raw sewage for viral gene fragments has become a popular method for detecting viral spread within a community [29,30,31]. A large number of studies have been conducted to correlate the viral gene copy levels in sewage with the clinical case counts in a community [29,30,31,32,33]. Wastewater-based epidemiology (WBE) has become a very powerful tool for informing the local health officials of any early signs of the community spread of COVID-19. Notably, WBE is not biased towards hospitalized individuals, as all infected individuals may shed viral RNA regardless of symptoms. When coupled with next-generation sequencing, WBE may be used to monitor the emergence of new viral variants. The clinical swab-derived RNA or number of clinical cases may not be informative due to quantitative and qualitative issues, and there are limited options for collecting additional samples from individuals [34,35]. Intriguingly, multiple studies have reported that patients with COVID-19 had altered gut microbiome, possibly due to dysregulated immune responses [36,37,38]. Hazan et al. [37] observed reduced levels of *Bifidobacterium*, *Faecalibacterium*, and *Roseburium* in patients with increasing severities of COVID-19. Gu et al. [36] reported that patients with COVID-19 had lower relative abundances of *Fusicatenibacter*, *Romboutsia*, and *Intestininbacter* and higher abundances of *Actinomyces* and *Erysipelatoclostridium* compared with healthy controls, and they suggested these five microbial genera as a set of biomarkers to differentiate between COVID-19 groups and controls. Thus, when there is an increase in individuals infected by COVID-19 within a community, the microbial diversity in wastewater could be impacted by the altered gut microbiomes of infected individuals. Subsequently, the changes in the microbial composition of wastewater could be tracked for additional biomarkers to strengthen current WBE approach with SARS-CoV-2. Overall, WBE has become an effective and convenient tool used by many local health authorities around the globe [28,39,40,41].

Although WBE has been advantageous in the COVID-19 pandemic, surveillance tools are often used for other infectious diseases such as poliovirus and other enteric pathogens. Given the strength of the technology, we initiated an effort to use the raw wastewater collected from the Toledo area and the University of Toledo’s (UToledo) dormitory areas to analyze the viral gene copies and sequence the new variants. Our results indicate not only correlative potential, but also identify new mutations that can affect viral replication. Moreover, the novel biomarkers may be applied to additional infectious diseases.

## 2. Materials and Methods

### 2.1. Ohio Wastewater Network and the University of Toledo Campus Monitoring Network

**Figure 1:**
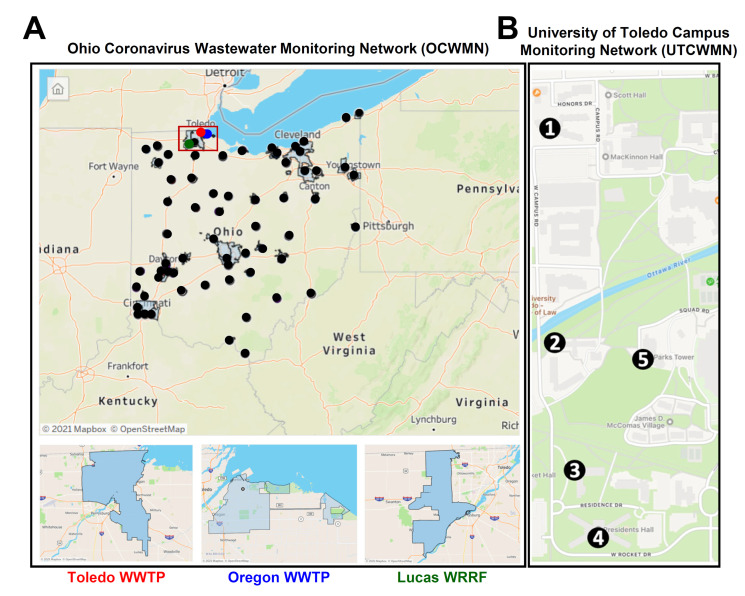
The geographical locations of the sample collection points. (**A**) A map of the Ohio Coronavirus Wastewater Monitoring Network (OCWMN), highlighting the sample collection points from the various WWTPs (top panel). The inset indicates the TOLWMN collection points. The WWTPs from the Toledo area used for the analyses by the OCWMN (bottom panel). (**B**) The UToledo campus map highlights the locations of the sample collection sites (1–5) in the UTCWMN.

To evaluate whether there is any correlation between the wastewater SARS-CoV-2 gene copies and the COVID-19 case counts, we began monitoring the SARS-CoV-2 viral gene copies in the local and campus raw wastewater. The Ohio Department of Health (ODH) created a wastewater network during the early onset of the COVID-19 pandemic to facilitate SARS-CoV-2 surveillance in the Toledo community. The Ohio COVID-19 Wastewater Monitoring Network (OCWMN) consisted of state university laboratories in Ohio, the US Environmental Protection Agency (EPA), and commercial laboratories. The goal of the network was to: (a) routinely monitor the SARS-CoV-2 gene copy levels in the raw sewage samples collected from wastewater treatment plants (WWTPs) from various parts of Ohio (Figure 1A), (b) inform the local healthcare system about any surge in the SARS-CoV-2 gene copies in the wastewater samples, (c) perform inter-laboratory cross-validation of split-samples, and (d) sequence wastewater-derived RNA to monitor variants of concern. The current study focused on the three Toledo area WWTPs (Toledo, Oregon, and Lucas County), as shown in Figure 1A, from the fall of 2020 to the spring of 2021. We refer to this project as the Toledo Oregon Lucas Wastewater Monitoring Network (TOLWMN). After the lockdown phase was relaxed and students were allowed to stay on the university campuses, the ODH initiated an independent project to monitor the raw sewage samples collected from the various university campuses. At the University of Toledo, we created the University of Toledo (UToledo) Campus Wastewater Monitoring Network (UTCWMN), which consisted of five sample collection areas, as shown in Figure 1B. Both networks routinely analyzed viral gene copy levels to correlate them with COVID-19 case counts.

### 2.2. Wastewater Sample Collection

Twenty-four-hour composite samples were collected using automatic sampling devices at the Toledo, Oregon, and Lucas county wastewater treatment plants (WWTPs, Figure 1A) once a week. The collected samples were transferred to screw-cap bottles and transported on ice to the laboratory for analysis. RNA was extracted within 24 h post-collection of the composite samples. For dormitories, a portable autosampler (AS950; Hach, Loveland, CO, USA) was used to collect composite samples into a 2.5-gallon bottle in each location (Figure 1B). We connected the autosampler to a suction tube with a ¾” inner diameter, which was attached to a standard weighted polypropylene strainer (Teledyne ISCO, Inc., Lincoln, NE, USA) that we used for all autosamplers. The tubes had varying lengths of 20–30 ft, depending on the depth required to reach the access points. Through the manholes, we placed the weighted steel strainers under the flowing wastewater stream to maximize wastewater collection. We programmed the autosamplers to obtain a composite sample over a 24 h period, with individual 20 mL pulls every 15 min. Approximately 300 mL of homogenized wastewater composite sample was collected every Monday and Wednesday in the morning from each location of the dormitories at the University of Toledo.

### 2.3. RNA Extraction from the Wastewater

The total RNA from the raw sewage samples was extracted by the RNeasy PowerWater Kit (QIAGEN Sciences, Inc., Germantown, MD, USA) using the manufacturer’s instructions and following the procedure described earlier [30,31]. The composite wastewater samples were processed in duplicate 180 mL volumes after adding 10× phosphate-buffered saline (PBS, Millipore-Sigma, Burlington, MA, USA). The buffered wastewater samples were centrifuged at 4000× *g* for 20 min, and the supernatants were filtered through 0.45 µm mixed cellulose ester membrane filters (Fisher Scientific, Hampton, NH, USA) to remove large debris. The membrane filters were aseptically transferred to the PowerWater bead tubes provided with the RNeasy PowerWater kit. The pellets were resuspended in the kit-supplied lysis buffer with β-mercaptoethanol (Millipore-Sigma) and then transferred to the PowerWater bead tubes containing the membrane filters. All PowerWater bead tubes were vortexed for 5 min and further processed according to the manufacturer’s instructions. The purified RNA was eluted from the spin filters with 100 µL of RNase-free water.

### 2.4. Quantitative RT-PCR Analyses of the SARS-CoV-2 Gene Copy Levels

The extracted RNA was reverse-transcribed by random hexamer primers using an ImProm-II Reverse Transcriptase Kit (Promega, Madison, WI, USA). The resultant cDNA library was diluted 1:2 using RNase-free water and analyzed by qPCR for the SARS-CoV-2 N2 gene, crAssphage, and MHV N using a Roche LightCycler 96 instrument (Roche, Basel, Switzerland). The total reaction mixture (10 μL/well), in duplicate wells, consisted of 5 μL of the Radiant Green Lo-ROX qPCR mix (Alkali Scientific Inc., Fort Lauderdale, FL, USA), 0.4 μL of primers (10 μM), and 4.6 μL of the diluted cDNA. The qPCR conditions were as follows: 95 °C for 2 min, 50 cycles of 95 °C for 5 s and 60 °C for 20 s, and 72 °C for 10 s. The serially diluted (20, 200, 2000, 20,000, and 200,000 copies/reaction) linearized SARS-CoV-2 N plasmid [42] generated the standard curves. The SARS-CoV-2 gene copy number per liter was calculated using the standard curve. To estimate the recovery efficiency, a murine hepatitis virus (MHV), obtained from BEI resources, was spiked in the PBS-buffered wastewater before the RNA extraction. The MHV RNA in the eluted RNA was analyzed by qRT-PCR using MHV N primers [43], and a gBlock containing the MHV N gene (IDT) was used as a standard control. Similarly, the crAssphage gene copy numbers were analyzed by qRT-PCR. For the inhibition test, the extracted RNA and the cDNA, at various dilutions (1:5, 1:10, 1:100, and 1:1000), were tested to amplify the unrelated gene fragments (e.g., murine interferon regulatory factors [44] or GFP).

### 2.5. SARS-CoV-2 Genome Amplification and Sequencing

The wastewater-derived RNA samples, selected based on relative abundance of SARS-CoV-2 gene copy levels, were resuspended in Trizol Reagent (Thermo Fisher, Waltham, MA, USA) at a ratio of 1 part sample to 4 parts Trizol Reagent. Four hundred microliters of the sample in Trizol Regent were extracted using the Direct-zol-96 MagBead RNA kit (Zymo Research, Irvine, CA, USA) following the manufacturer’s standard protocol, including the optional DNAse treatment. The RNA extracted from each sample was converted to cDNA, and the SARS-CoV-2 genome was enriched using multiplexed tiling PCR-based target amplification using the ARTIC 400 bp primer set [45]. Illumina sequencing of the PCR products was used to obtain sequencing data spanning the SARS-CoV-2 genome. To generate cDNA, 1 μL 50 mM random hexamer primer, 1 μL 10 mM dNTPs, and 11 μL of extracted RNA were mixed and cycled for 5 min at 65 °C. All reactions were placed on ice for 1 min. An addition of 4 μL SuperScript IV buffer (Thermo Fisher), 1 μL 100 mM DTT, 1 μL RNAseOUT (Thermo Fisher), and 1 μL SuperScript IV enzyme (Thermo Fisher) was made to each reaction to bring the total volume to 20 μL per reaction. All reactions were cycled for 50 min at 42 °C, 10 min at 70 °C, and held at 4 °C. Following cDNA generation, all samples were amplified using the ARTIC Network SARS-CoV-2 400 bp amplicon V3 primer set [45]. Each reaction consisted of 5 μL 5× Q5 Reaction Buffer (New England Biolabs, Ipswich, MA, USA), 0.5 μL 10 mM dNTPs, 0.25 μL Q5 Hot Start High Fidelity DNA polymerase (New England Biolabs), 4.0 μL primer pool (Integrated DNA Technologies, Coralville, IA, USA), 12.75 μL water, and 2.5 μL cDNA reaction. Separate reactions were done for each of the 2 primer pools for the ARTIC 400 bp primer set. Each reaction was cycled for 30 s at 98 °C, followed by 35 cycles of 15 s at 98 °C and 5 min at 65 °C.

The 2 completed PCR reactions (pool 1 and 2) were combined for each sample for a total volume of 50 μL. Each PCR reaction pool was purified by adding 0.8× (40 μL) Ampure XP beads (Beckman Coulter, Brea, CA, USA). The standard manufacture protocol was followed, and following 2.75% ethanol washes, the samples were resuspended in 25 μL water. The purified samples were examined on an Agilent Tapestation 4000 using the D1000 ScreenTape, and the appropriately sized product was confirmed in each sample. The Nextera Flex library kit (Illumina, San Diego, CA, USA) was used to construct sequencing libraries from the purified amplicon samples using the manufacturer’s standard protocol for small amplicons. A total of 5–10 ng of each sample was input into the library preparation procedure. The DNA concentrations of the completed libraries were determined using a broad range dsDNA kit on a Qubit fluorimeter (Thermo Fisher). All libraries were diluted to 4 nM, pooled, denatured, and diluted to a loading concentration of 1.3 pM, per the manufacturer’s instructions. The 1.3 pM pooled libraries with 1% phiX control were loaded onto an Illumina NextSeq 500 instrument using a Mid Output kit version 2.5.

### 2.6. Bioinformatic Analysis

The sequencing reads (FASTQ) were annotated into variant calls using mappgene (https://github.com/LLNL/mappgene, version v1.2.1, accessed on 10 January 2022), a modular bioinformatics pipeline for high-performance computing developed at the Lawrence Livermore National Laboratory (LLNL) for highly sensitive variant detection. Mappgene employs BWA-MEM for the read alignment, iVar for read trimming/filtering/variant calling [45], and LoFreq [46] to call variants.

### 2.7. Microbiome Analyses

From the wastewater samples collected, bacterial genomic DNA was extracted using the DNeasy PowerSoil pro kit (Qiagen, Germantown, MD, USA) [47]. Then, the 16S rRNA gene library was constructed using the primer set 515f-806r to target the V4 variable region suggested by the Earth Microbiome Project (http://www.earthmicrobiome.org, accessed on 29 November 2021). The Illumina NovaSeq platform was employed to generate 150 bp long sequences. DNA extraction, library preparation, and sequencing work were performed at the Genomics Facility at the Biodesign Institute of Arizona State University. QIIME2 (Quantitative Insights Into Microbial Ecology, version 2) software was employed for the microbiome analysis [48]. Through the QIIME2 DADA2 plugin pipeline [49], we obtained 707,156 sequence reads in total with good quality, and taxonomy were assigned to feature sequences using a naive Bayes classifier pre-trained by the Greengenes database. The relative abundances of all bacterial genera detected across the samples were tested with SARS-CoV-2 gene copy numbers by a non-parametric Spearman correlation test. After correcting for multiple testing by p.adjust in R programming, the adjusted *p*-values of less than 0.05 were accepted as significant.

## 3. Results and Discussion

**Figure 2:**
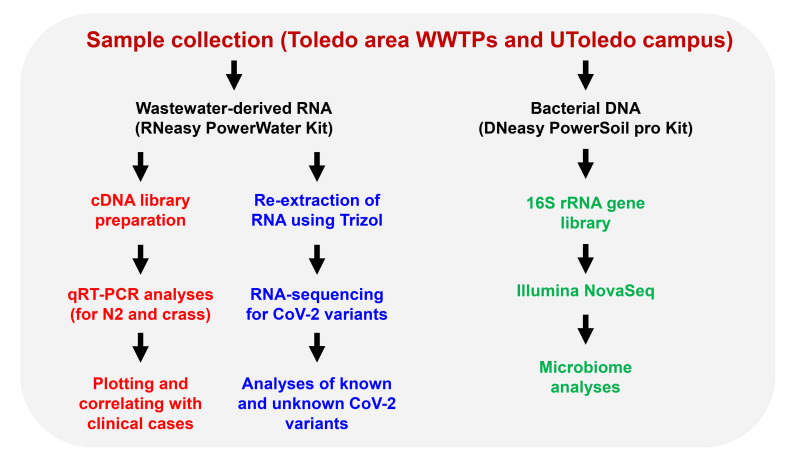
An overview of the Toledo area wastewater surveillance for COVID-19. Raw sewage samples were collected from Toledo area WWTPs and the UToledo campus and were used for RNA extraction. The RNA isolated from the wastewater samples were analyzed using qRT-PCR and the RNA was further analyzed by high-throughput sequencing for the identification of SARS-CoV-2 variants. The sewage samples were also used for microbiome analyses by extracting the bacterial genomic DNA, as shown.

The state (OCWMN) and the campus monitoring projects aimed to analyze the raw wastewater samples collected from either the WWTPs or the university campus for the levels of SARS-CoV-2 viral gene copies. The viral gene copy levels in the wastewater were then correlated with the COVID-19 case numbers in the community using statistical models to determine current and near-future community spread. Furthermore, we performed high-throughput RNA-sequencing (RNA-seq) analyses of the wastewater-derived total RNA to determine the SARS-CoV-2 viral mutants and variants, both clinically known and unknown (Figure 2).

### 3.1. Correlation between the Wastewater SARS-CoV-2 Viral Gene Copies and the Case Counts

**Figure 3:**
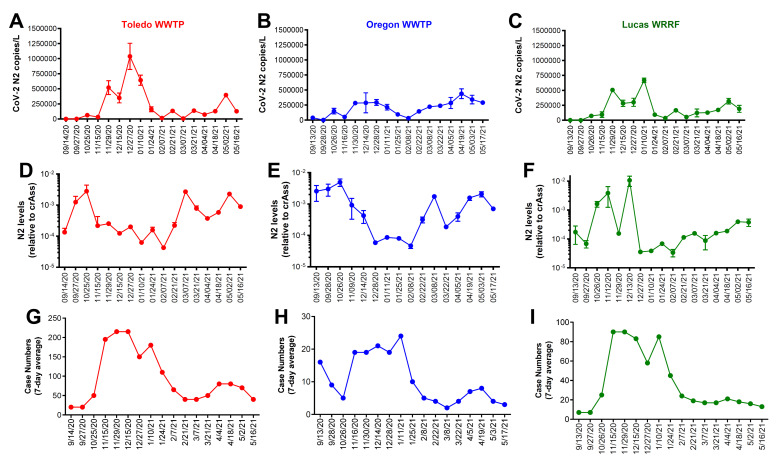
The analyses of the SARS-CoV-2 gene copies in the samples collected from the Toledo area WWTPs in the TOLWMN program. (**A**–**C**) SARS-CoV-2 viral N mRNA levels (copies/L) were analyzed by qRT-PCR. (**D**–**F**) The levels of SARS-CoV-2 viral N mRNA relative to the crAssphage. (**G**–**I**) The 7-day average case numbers in the Toledo area were obtained from the ODH website.

**Figure 4:**
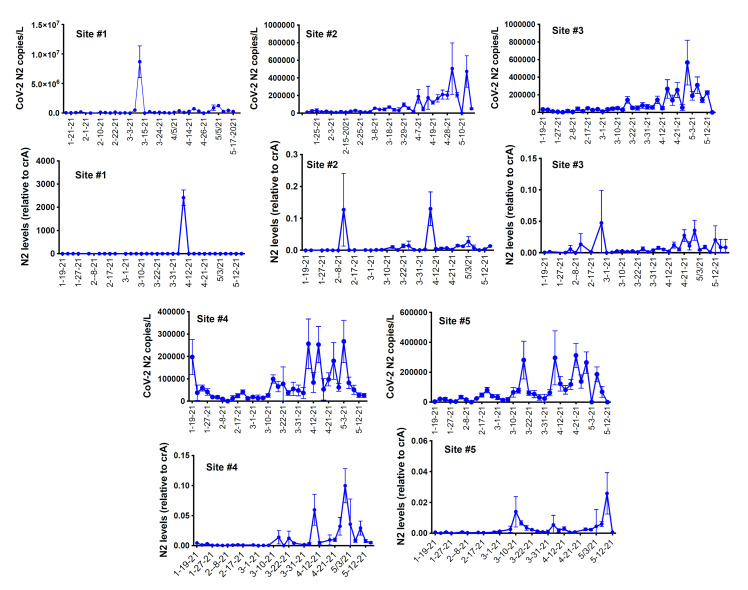
The analyses of the SARS-CoV-2 gene copies in the samples collected from the UToledo campus sites in the UTCWMN program. The SARS-CoV-2 viral N mRNA levels (copies/L), relative to crAssphage, were analyzed by qRT-PCR.

**Figure 5:**
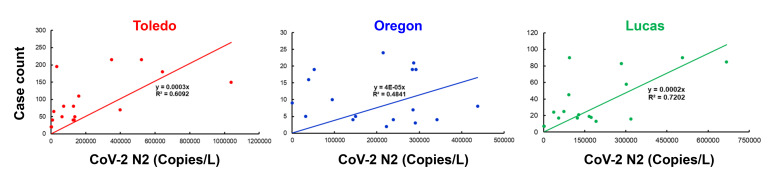
The correlative analyses of the SARS-CoV-2 gene copies with the COVID-19 case counts. The gene copies (per L) were plotted against the case numbers and correlative analyses were performed.

**Figure 6:**
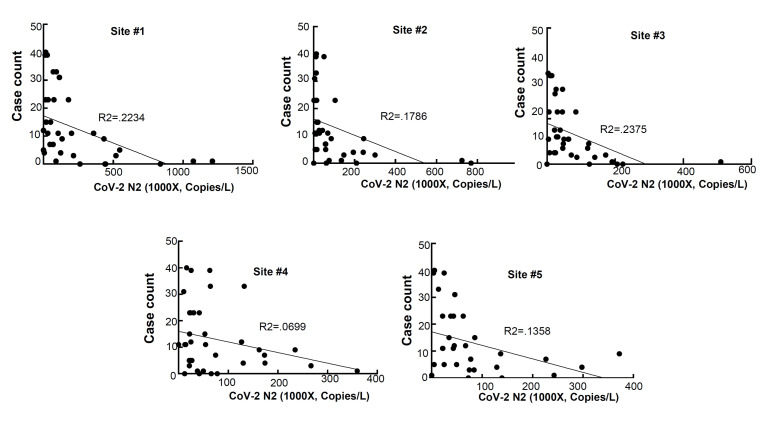
The correlative analyses of the SARS-CoV-2 gene copies with the COVID-19 case counts. The gene copies (per L) were plotted against the case numbers and the correlative analyses were performed.

RNA was extracted from raw sewage collected from the Toledo area WWTPs and subsequently assayed by qRT-PCR for the SARS-CoV-2 gene copies (viral N gene) and a human marker (crAssphage), as described in the Methods. From the UTCWMN program, the SARS-CoV-2 viral gene copies were plotted against the time of collection (Figure 3A–C). Similarly, we plotted the crAssphage-normalized SARS-CoV-2 viral gene copies, i.e., the SARS-CoV-2 gene copies relative to crAssphage, against the time of collection (Figure 3D–F). Finally, the case counts (7-day average, data obtained from the ODH website) were plotted for the time range that was used for the wastewater analyses (Figure 3G–I). Similar to the TOLWMN analyses, the samples collected for the UTCWMN program were also analyzed for viral gene copies and crAssphage-normalized viral gene copies (Figure 4). There are a number of key observations that we made from these analyses: (a) the trends of the SARS-CoV-2 gene copy (N2, SARS-CoV-2 N gene copies, analyzed by using the CDC N2 primers) levels were different from those of the N2/crAssphage (N2/crass) in both the TOLWMN and UTCWMN programs; (b) in the TOLWMN analyses, we visually observed a relatively better correlation between the N2 levels with the clinical cases. Next, we performed direct correlative analyses between the case counts with either the viral gene copy levels (N2) or the normalized gene copy levels (N2/crass) for both the TOLWMN and UTCWMN programs. We observed a potential correlation between the clinical COVID-19 case counts and the gene copy levels, which was stronger for the Toledo and Lucas sites (Figure 5). Similarly, a correlation between the clinical case counts and the viral gene copies was also observed for the UTCWMN sites (Figure 6). Together, these results demonstrate a strong correlation between the wastewater gene copies and the clinical case counts.

### 3.2. Sequence Analysis of the Wastewater Samples

**Figure 7:**
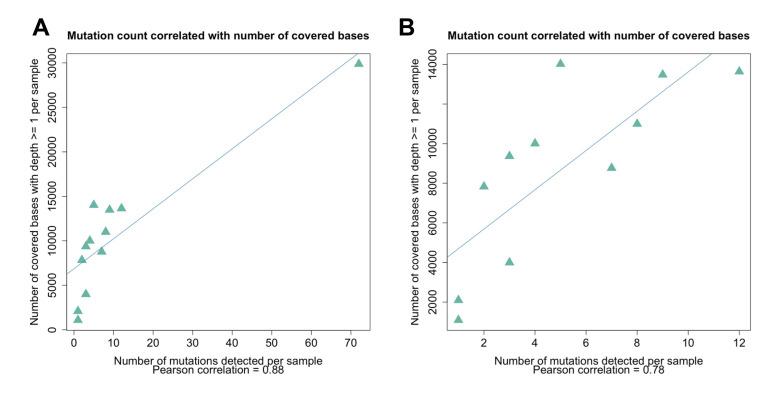
Correlation between the genome coverage depth and the number of mutations detected. (**A**) The mutation count was correlated with the number of bases (nucleotide positions) covered by one or more reads (Pearson correlation of 0.88). (**B**) The removal of the sample that had extremely high coverage and a large number of mutations detected (Lucas 122020) resulted in a lower but still significant correlation (Pearson correlation of 0.78).

**Figure 8:**
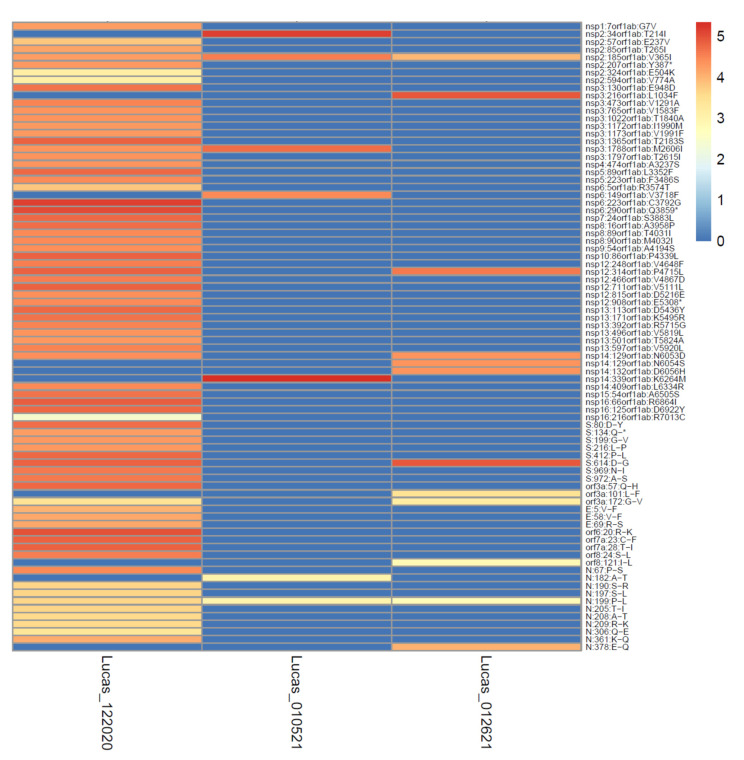
High depth of coverage across the genome increases the sensitivity of mutation detection. The data from three samples from Lucas collected in the winter of 2020–2021 varied according to depth of coverage (the colors of the cells depict the logarithm of the number of reads covering each mutation site) and mutations detected (mutation sites shown on the *y*-axis).

The depth of the sequencing read coverage and the percent coverage across the genome varied between the samples, and the samples from the UToledo dormitories had significantly fewer reads generated, potentially due to differences in sludge processing or the presence of unknown inhibitors [50]. The extent of the sequencing coverage impacted the sensitivity of the variant detection (Figure 7 and Figure 8), and the percentage of the genome covered was positively correlated with the number of mutations detected (Pearson correlation of 0.88; Figure 7). The correlation between the number of mutations detected per sample and the mean depth per sample was moderate (Pearson correlation of 0.54). All samples except for Lucas 122020 had less than half the genome with coverage (reads mapped), whereas Lucas 1220220 had reads mapped across almost the entire genome, and the majority of the mutations were detected in this sample (Figure 7A). The spike D614G mutation became dominant within the first few months of the pandemic [51]; however, this mutation was detected in only 3 of the 64 samples sequenced (Lucas 122020 and 012621 and Toledo 010521) (Table 1). Analysis of the coverage for each sample showed that there was no coverage at the spike residue 614 for the other Lucas and Toledo area samples.

The wastewater samples were collected from the four different locations between the end of November 2020 and the beginning of June 2021, and the later sampling dates overlapped the emergence of variants of concern (VOC) and variants of interest (VOI) in the US and Ohio (outbreak.info variant emergence Supplementary Figure S1). To determine if any of these variant genotypes were circulating in the Toledo area, the sequence data were checked for the presence of mutations that characterized the VOC and VOI genotypes. Nine mutations associated with the VOC and VOI circulating during that timeframe were detected (Table 1). Most of these mutations had already emerged early in the pandemic and were well-established in multiple clades prior to the emergence of the VOC and VOI in December 2020. Analysis of the sequence data focused on the recently emerged, “signature mutations” of the VOC and VOI, such as the spike mutations N501Y and E484K that emerged late 2020 and early 2021.

Three signature mutations were detected, and all occurred in the N protein. The mutations affected residues 199, 205, and 235, which fall within the linker region of the protein (residues 174 to 249) [52]. The N protein mutation S235F is distinct for the Alpha VOC and was detected in the sequence data from Oregon collected in mid-May 2021 (Oregon 051721), with >99% of the reads showing this mutation. By mid-May, the Alpha variant was well-established in Ohio at a case prevalence of approximately 80% (Supplementary Figure S1), and thus the detection of S235F in this sample agrees with the timing of the Alpha variant’s emergence in Ohio.

The T205I mutation in the N protein is present in multiple VOC and VOI (Beta, Mu, Iota, and Epsilon), and it was detected in 9% of the sequencing reads from a sample from the Lucas location (Lucas 122020) collected in mid-December. Data from outbreak.info (Supplementary Figure S2) show that the prevalence of this mutation peaked in the US in early 2021 and was detected in slightly less than 10% of cases in mid-December 2020, and thus this mutation was detected by wastewater analysis relatively early.

Nucleoprotein mutation P199L was first detected in clinical samples early in 2020 and is part of the Iota VOI genotype, but it did not peak in the US until much later. This mutation was detected in four samples from Toledo (010521) and Lucas (122020, 010521, and 012621) in December and January at the peak of the mutation’s prevalence in the US (Supplementary Figure S2).

The exceptionally high depth of read coverage (24599x) generated for the Lucas sample 122020 enabled the detection of a deletion at the spike protein residue 144 at a very low frequency (0.13%). This deletion is characteristic of the Alpha VOC and may disrupt the antibody binding [53]. These data illustrate the potential sensitivity of the high depth and coverage sequence data for the detection of emergent variant mutations in wastewater. As depicted in Figure 8, the high depth of read coverage across the genome enabled many more mutations to be detected in the Lucas sample 122020, even as compared to other Lucas samples from similar collection times.

Although the fragmented nature of viral RNA in wastewater samples may preclude the detection of intact genotypes, a combination of mutations present within samples may be indicative of shifting of clades within a region over time. Indeed, a combination of mutations detected in the samples from this study are characteristic of the 20G clade, a clade which was described in a previous study [54] to be present in clinical samples collected in Columbus, Ohio from April 2020 through January 2021 and representing a shift from the 20C clade. For example, six of the eight mutations that define the backbone of the 20G clade [54] were detected in the Lucas 122020 sample. Similarly, 10 of the 10 mutations that characterize the 20G clade, as described by a genome surveillance study of samples from New Mexico and Louisiana [55], were detected in one Lucas sample (Lucas 122020) and a total of seven other samples from Lucas, Toledo, and Oregon had one to four of these mutations. Notably, each of these studies mentioned the presence of distinct mutations at the spike protein residues (501 and 677), which were also present in the 20G clade samples, but neither of these mutations were detected in any of these samples from the Toledo area. These data demonstrate the ability of the genetic surveillance of wastewater samples to detect the presence of emerging lineages even in the absence of key mutations that may not be detected due to genome fragmentation and/or geographic differences in clade genotypes [55].

A relatively rare mutation, V365I (gene orf1a) was detected in three Lucas samples (Lucas 122020, 010521, and 012621) between 20 December 2020 and 26 January 2021. This mutation was not associated with emergent variants (the VOC and VOI) and was seldom detected in US samples, with a total of 1481 US sequences reported as showing this mutation (as reported by outbreak.info) from July 2020 to October 2021, with a peak of approximately 1% (Supplementary Figure S3A). However, this particular mutation was detected at a relatively high prevalence in Ohio during the time period that the wastewater samples were collected, with 294 sequences reported from November 2020 to July 2021 and a peak of approximately 20% (Supplementary Figure S3B). The mutation was detected at a high frequency in the Lucas 122020, 010521, and 012621 wastewater samples, with 99.95% of the Lucas 122020 and 010521 reads containing the mutation and 55.93% of the reads from Lucas 012621 showing the mutation. Each of the samples with this mutation detected had a high sequence coverage at this site (range 7178–35,661 × coverage). This mutation was located within the second zinc finger of the nsp2 protein [56], and the structural impact is unknown. These data indicate that wastewater sequence data with a high coverage and depth can reflect regional trends of mutation emergence in clinical samples.

### 3.3. Potential Bacterial Biomarkers to Strengthen Wastewater SARS-CoV-2 Surveillance

**Figure 9:**
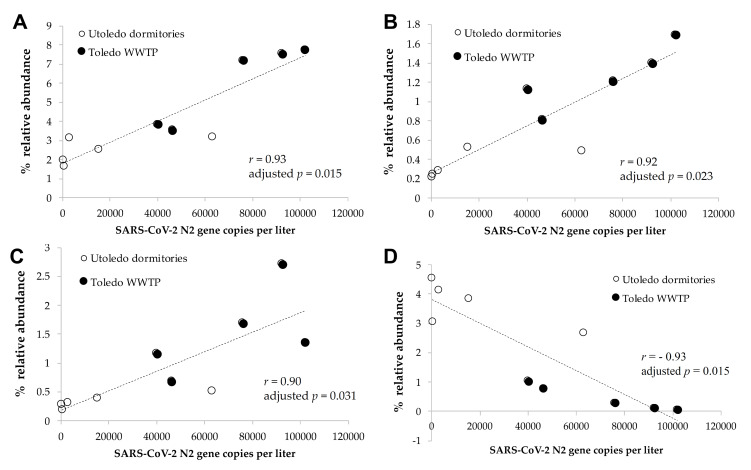
The correlation of SARS-CoV-2 gene copies with microbiome markers. There were significant correlations between the numbers of SARS-CoV-gene copies and the relative abundances of the bacterial genera (**A**) *Blautia*, (**B**) *Coprococcus*, (**C**) *Roseburia*, and (**D**) unclassified *Actinomycetaceae* in the wastewater samples (Spearman correlation of r > 0.90, adjusted *p* < 0.05).

Patients with COVID-19 have a disturbed and less-diversified gut microbiome due to increased gut inflammation [36,37,38]. A patient’s gut microbiome could alter microbial profiles in wastewater when their feces are shed into that wastewater together with SARS-CoV-2 genes [29,32,33]. Thus, we hypothesized that an increase in COVID-19 cases could alter microbial profiles, especially the bacterial composition, in wastewater, which would have a similar trend with the changes in the number of SARS-CoV-2 genes. Then, these additional microbial biomarkers could be leveraged as prediction tools for future infectious disease surveillance and outbreak responses to strengthen the current WBE approach. To test this hypothesis, we profiled bacterial communities in 10 wastewater samples by sequencing the 16S rRNA genes of bacteria, and we investigated correlations between the amounts of SARS-CoV-2 genes and the relative abundances of individual bacterial genera. The most dominant bacterial genera were *Acinetobacter*, *Acidovorax*, *Bifidobacterium*, and *Blautia*, and the detailed information on the dominant genera and families can be found in the Table S1. We observed strong positive correlations between the relative abundance of the genera *Blautia*, *Coprococcus*, *and Roseburia* (all of these genera are of the family *Lachnospiraceae*) and the number of SARS-CoV-2 gene copies in the wastewater (Spearman correlation of r > 0.90, adjusted *p* < 0.05, Figure 9A–C). This result is intriguing since recent studies reported that those genera (*Blautia*, *Coprococcus*, and *Roseburia*) were reduced in the guts of patients with COVID-19 (regardless of their antibiotic usages) [38,57], and the presence of the genus *Roseburia* was negatively correlated with the degree of COVID-19 severity [58]. The genus *Blautia* was the second-most enriched genus in the gut of recovered patients [59] and illustrated the strongest significant positive correlation with SARS-CoV-2 genes in wastewater in our study. The genera listed above are known as potentially beneficial bacteria [60,61,62], and healthy subjects may lose them when infected with COVID-19. These bacteria could be shed into wastewater, together with COVID-19, which would explain the positive correlation between the presence of those bacterial genera and the SARS-CoV-2 genes that we observed in our wastewater samples. Meanwhile, we observed that the *Actinomycetaceae* taxon was negatively correlated with the SARS-CoV-2 gene copies in wastewater (Spearman correlation of r = −0.93, adjusted *p* < 0.05, Figure 9D). Zuo et al. [58] reported that *Actinomyces viscosus*—an opportunistic pathogen—in the genus *Actinomyces* was enriched in the feces of COVID-19 patients, and was positively correlated with the severity of illness. Although it needs to be validated with extended wastewater samples, the bacterial genera above could serve as biomarkers to support SARS-CoV-2 surveillance in wastewater and better predict a re-emergence of an outbreak.

## 4. Conclusions

Wastewater-based epidemiology has been used extensively as a predictive tool for the COVID-19 outbreak globally. Our results from the Toledo area provide a correlative potential between the viral gene copies and the COVID-19 clinical case counts. The RNA-seq analyses, in combination with viral load, added strengths to our study for the identification of new viral variants. Continuous and frequent sampling, analyses of wastewater for viral gene copies, and sequencing may be a way forward to effectively manage the pandemic in the future. Among the various challenges associated with WBE-based studies is the variation in results due to different extraction and analytical methods. Efforts should be directed toward universal RNA extraction methods, either directly or by concentrating the raw sewage, to compare the results efficiently and to be used as a global predictive tool. Moreover, frequent high-throughput sequencing of wastewater-derived RNA should be performed to identify new viral variants and test them for virological studies. Our study further suggests that bacterial biomarkers can be used as an independent correlative tool for viral gene copies. Finally, the wastewater-based epidemiological tools can be expanded to other infectious diseases, e.g., an influenza epidemic, for better management of the public health system.

## Figures and Tables

**Table 1:** Sample count and frequency of mutations present in the VOC and/or VOI genotypes.

Sample Count	Gene	AA Pos	Ave Alt Freq	Med Alt Freq	Ref AA	Alt AA	Toledo	Lucas	Oregon	UT
4	orf1ab	265	100.0%	100.0%	T	I	0	1	3	0
4	orf1ab	314	100.0%	100.0%	P	L	2	2	0	0
3	S	614	100.0%	100.0%	D	G	1	2	0	0
4	orf3a	57	100.0%	100.0%	Q	H	3	1	0	0
4	N	199	97.6%	99.8%	P	L	1	3	0	0
1	N	203	99.9%	99.9%	R	K	0	0	1	0
1	N	204	100.0%	100.0%	G	R	0	0	1	0
1	N	205	9.1%	9.1%	T	I	0	1	0	0
1	N	235	99.9%	99.9%	S	F	0	0	1	0

Sample Count: number of samples with the mutation; AA Pos: residue number in the gene with the amino acid mutation; Ave Alt Freq: average frequency of the alternate amino acid (mutation); Med Alt Freq: median frequency of the alternate amino acid; Ref AA: amino acid in the reference sequence; Alt AA: amino acid in the sample sequence.

## Data Availability

The microbiome sequencing datasets supporting the conclusions of this article are available in the open-source microbiome database “Qiita” with the study ID number 14752 (https://qiita.microbio.me) for 16S rRNA gene sequence reads.

## Supplementary Materials

The following supporting information can be downloaded at: https://www.mdpi.com/article/10.3390/v14092032/s1, Figure S1: Mutation and case prevalence of emergent genotypes in Ohio as depicted on outbreak.info; Figure S2: Emergence of N protein mutation T205I in the US; Figure S3: Emergence of N protein mutation P199L in the US; Figure S4: Prevalence of mutation orf1a V365I in sequences from the (A) US and from (B) Ohio; Table S1: Detailed information on the dominant genera and families.
