# Supplementary material for: SARS-CoV-2 Monitoring in Wastewater Reveals Novel Variants and Biomarkers of Infection
Source: Viruses. 2022 Sep 13;14(9):2032. doi: 10.3390/v14092032 (PMC9503862; doi:10.3390/v14092032)
Supplement: Supplementary file 1 [file viruses-14-02032-s001.zip › Suppl doc.pdf]

Supplemental information

Primer sequences:

N2-fwd: TTACAAACATTGGCCGCAAA
N2-rev: GCGCGACATTCCGAAGAA

Crassphage-fwd: CAGAAGTACAAACTCCTAAAAAACGTAGAG
Crassphage-rev: GATGACCAATAAACAAGCCATTAGC

MHV-fwd: GCCAAATAATCGCGCTAGAA
MHV-rev: CCGAGCTTAGCCAAAACAAG

qRT-PCR standard details:

Y-intercept: 36.55
Slope: 2.923
LOD: 850 copies/L
LOQ: 1705 copies/L
