# Supplementary material for: SARS-CoV-2 Monitoring in Wastewater Reveals Novel Variants and Biomarkers of Infection
Source: Viruses. 2022 Sep 13;14(9):2032. doi: 10.3390/v14092032 (PMC9503862; doi:10.3390/v14092032)
Supplement: Supplementary file 1 [file viruses-14-02032-s001.zip › 070522_WW Suppl figures.pdf]

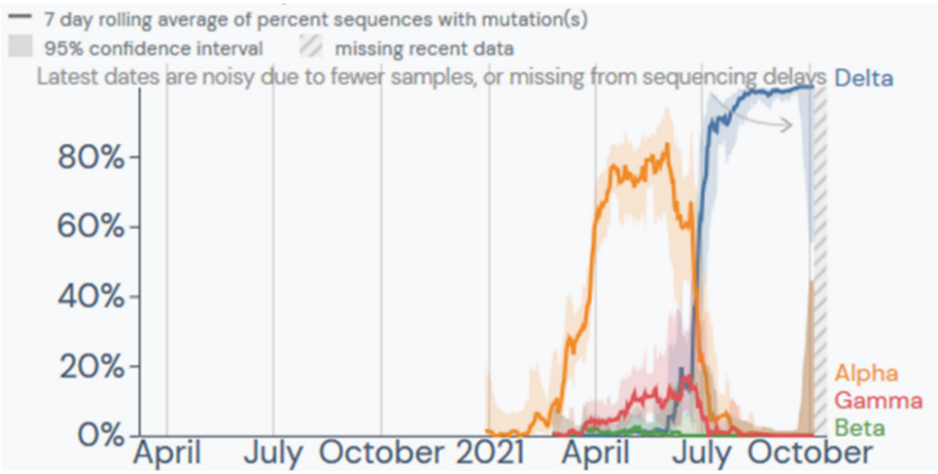

Suppl Fig 1. Mutation and case prevalence of emergent genotypes in Ohio as depicted on outbreak.info. The graph depicts the 7 day rolling average of percent positive sequences for each variant. The colored shading shows the 95% confidence interval.

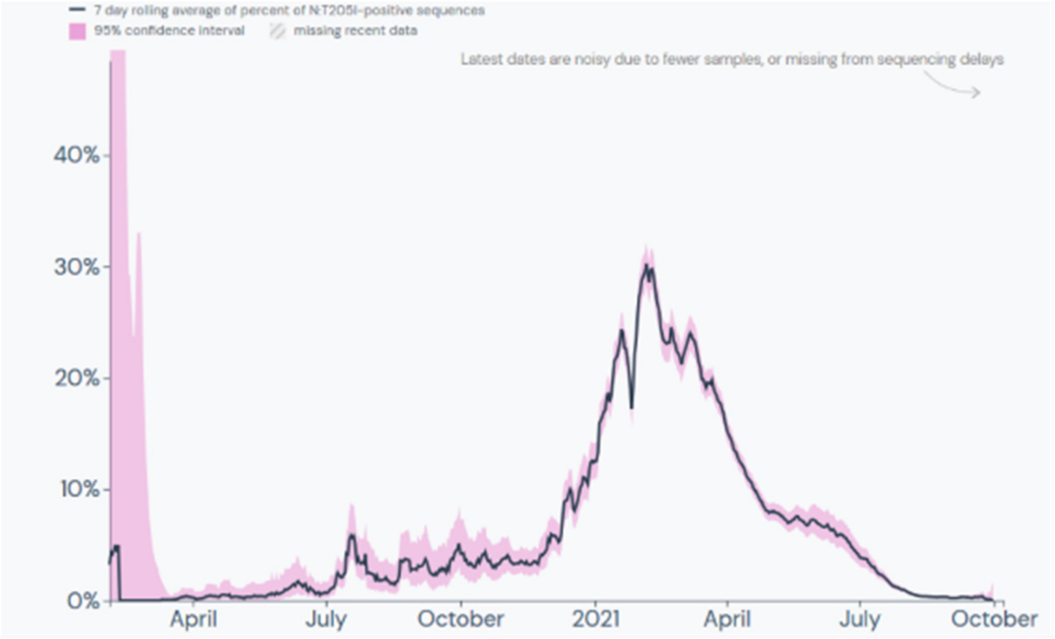

Suppl. Fig. 2. Emergence of N protein mutation T205I in the US. The graph generated using outbreak.info shows the prevalence of this mutation peaked over the time period that wastewater was sampled. The graph depicts the 7-day rolling average of percent positive sequences. The pink shading shows the 95% confidence interval.

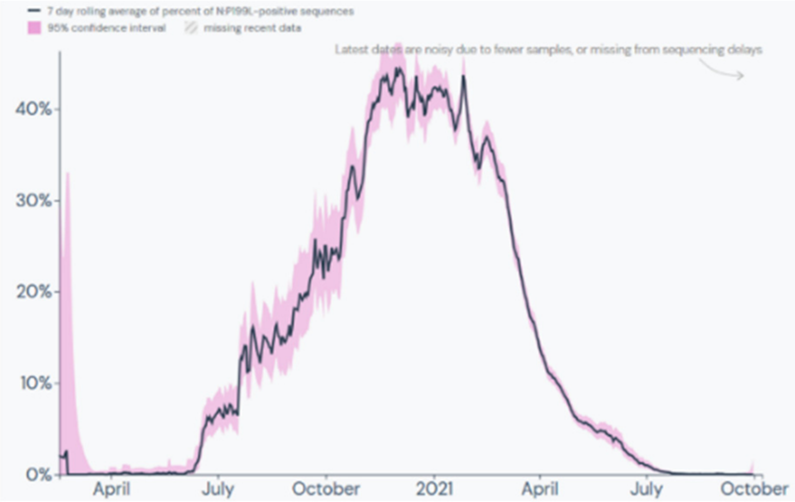

Suppl. Fig. 3. Emergence of N protein mutation P199L in the US. The graph generated using outbreak.info shows the prevalence of this mutation peaked over the time period that wastewater was sampled. The graph depicts the 7-day rolling average of percent positive sequences. The pink shading shows the 95% confidence interval.

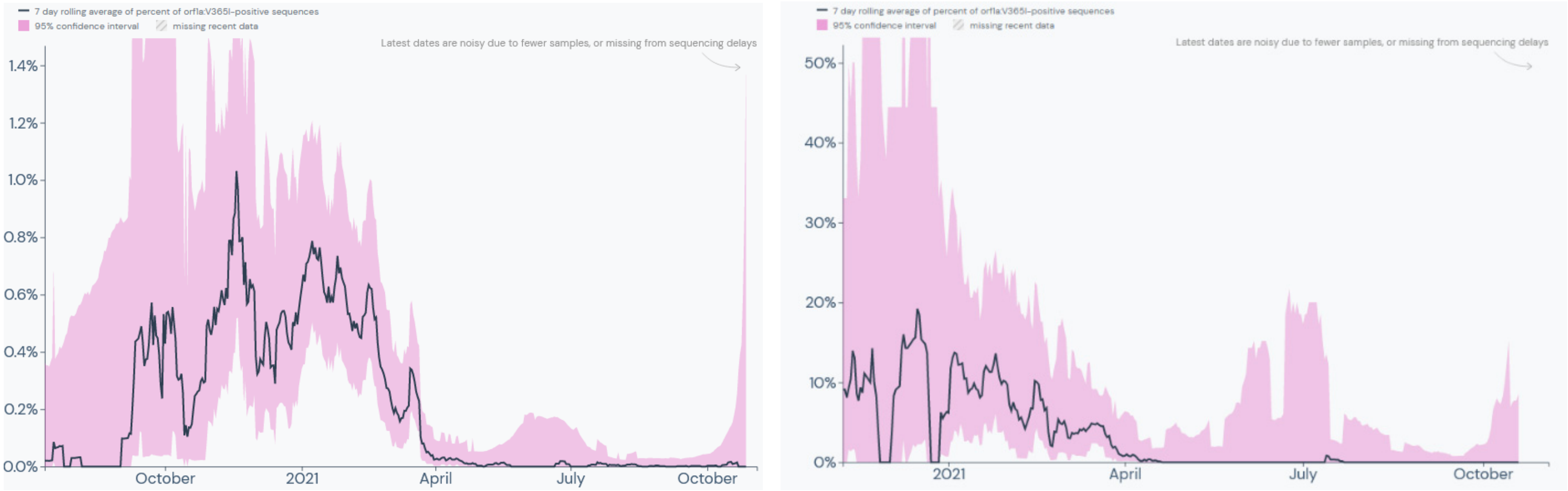

Suppl. Fig. 4. Prevalence of mutation orf1a V365I in sequences from the (A) US and from (B) Ohio. Comparison of the prevalence of the mutation in US versus Ohio sequences show the V365I mutation is detected at higher percentage of samples in Ohio during the timeframe that the wastewater samples were collected (note the y axis scale differs between graphs). The gaps in 2020 data from Ohio likely reflect that low numbers of samples were sequenced at some of these dates decreasing sensitivity of detection. The graphs were generated using outbreak.info. The graph depicts the 7 day rolling average of percent positive sequences. The pink shading shows the 95% confidence interval.
